# Ocular manifestations of COVID-19: facts and figures from a tertiary care center

**DOI:** 10.1080/07853890.2022.2029554

**Published:** 2022-01-21

**Authors:** Nissar Shaikh, Huda Al Mahdi, Anant Pai, Ankush Pathare, Ahmad A. Abujaber, Ashwin Dsliva, Mahmood Al-Jabry, Kumran Subramanian, Saju Thomas, Ahmed S. Mohmed, Shahzad Anjum, Abdulqadir J. Nashwan, Mohammad Al Wraidat, Mohamad Y. Khatib

**Affiliations:** a Surgical Intensive Care Department, Hamad General Hospital (HGH), Hamad Medical Corporation (HMC), Doha, Qatar; b Ophthalmology Department, Hamad General Hospital (HGH), Hamad Medical Corporation (HMC), Doha, Qatar; c Accident and Emergency Department, Hazm Mebaireek General Hospital (HMGH), Hamad Medical Corporation (HMC), Doha, Qatar; d Accident and Emergency Department, Hamad General Hospital (HGH), Hamad Medical Corporation (HMC), Doha, Qatar; e Critical Care Department, Hazm Mebaireek General Hospital (HMGH), Hamad Medical Corporation (HMC), Doha, Qatar

**Keywords:** COVID-19, comorbidities, inflammatory markers, ocular manifestations, hyperaemia

## Abstract

**Introduction:**

COVID-19 patients presenting with ocular manifestations are from 0.8% to 32% of patients seen in the ED. The available literature is scarce regarding COVID-19 patients presenting with ocular manifestations from the Middle Eastern region.

**Purpose:**

This study aims to report the incidence of ocular signs and symptoms in COVID-19 patients and find any correlation between the occurrence of ocular manifestations and patients’ comorbidities.

**Methods:**

All patients having the primary diagnosis of COVID-19 infection and concurrent ocular manifestations on admission to our tertiary COVID-19 health care centre were included in the study. The patient’s demographic data, comorbidities, and type of ocular manifestations were recorded from the patients’ health records retrospectively.

**Results:**

In our study, 39 (7.8%) patients presented with ocular manifestations. The majority of COVID-19 patients were male, and 200 (20%) patients had a history of other comorbidities. The majority of our patients had hyperaemia (13 [33.3%]), followed by eye pain (9 [23.1%]), epiphora (8 [20.5%]), burning sensation (4 [10.3%]), and photophobia (2 [5.1%]) patients. There was no statistically significant difference in the occurrence of ocular manifestations and patients’ gender or comorbidities (*p* > .05).

**Conclusion:**

The occurrence of ocular manifestations was lower compared to the present literature. There was no significant association between the occurrence of ocular manifestations and the patient’s gender or comorbidities.

## Introduction

COVID-19 pneumonia was initially reported in China in late 2019. Despite aggressive restrictive and preventive measures, it quickly spread to become a global pandemic, greatly impacting peoples’ lives, finances, and the world economy [1]. Since COVID-19 pneumonia is a potentially life-threatening condition, the primary focus of the research has naturally been on the respiratory system. But the COVID-19 infection can involve several other organs of the body, and there is growing evidence of its impact on eyes and vision. Ocular manifestations in COVID-19 patients can vary. Significant restrictions and reduction may hinder the documentation of ocular symptoms and signs in patients with COVID-19 infection in ophthalmic services during the ongoing pandemic [2]. In addition, it is understandable that the care and observations of the life-threatening conditions take precedence over the ocular conditions and their clinical documentation [2].

The prevalence of ocular symptoms and signs in COVID-19 patients can range from <1% to >30% [3,4]. Initial reports from China reported around 0.8% of all COVID-19 patients had signs and symptoms of eye involvement. The various ophthalmic features in COVID-19 patients described in the literature include burning eyes, foreign body sensation in eyes, photophobia, clear watery discharge, eyelid edoema, conjunctival hyperaemia, conjunctival injection, pseudomembranous and haemorrhagic conjunctivitis, follicular kerato-conjunctivitis and corneal sub-epithelial infiltrates [5,6]. It is also been reported that, rarely, ophthalmic features may represent the first clinical presentation in patients with COVID-19 infection [5,6]. More recent data suggest that ocular manifestations are higher than initially reported and are up to 11.03% in some studies [7].

There are no publications or available literature from the Middle East region on this topic. Therefore, the purpose of our study was to report the occurrence of ocular manifestations and the impact of comorbidities in COVID-19 patients in a tertiary care setup.

## Methods

After obtaining permission from the medical research centre of our hospital, all patients presented from March to May 2021 with ocular manifestation with COVID-19 infections confirmed by throat polymerase chain reaction (PCR) were included retrospectively in this study. All patients’ demographic data, comorbidities, details of ocular symptoms were recorded from the patients’ electronic files retrospectively.

Data were entered and analyzed using IBM SPSS version 23. Descriptive statistics in the form of mean and standard deviation were performed for interval variables. The frequency with percentages was calculated for categorical variables. Chi-square tests were performed to determine the association between categorical variables. Student’s *t*-test was performed to determine statistically significant mean differences between interval variables, the *p*-value of ≤.05 (two-tailed) was considered statistically significant.

## Results

During the study period, a total of 500 patients were admitted with COVID-19 pneumonia to our tertiary care centre. Only 39 (7.8%) patients had ocular manifestations at the hospital’s presentation time (Table1). The majority of patients (463 [92.6%]) were male. 200 (40%) patients had various comorbidities, including hypertension, diabetes mellitus, coronary artery disease, rheumatic arthritis, or cancer. CT value was less than 30 in 94.2% of patients. The majority of patients had normal leucocytes and CRP (C - reactive protein) 477 (95.4%) and 263 (52.6%), respectively (Table 1).

**Table 1.** Patients’ demographics.

Variable	Number	Percentage
Gender		
Male	463	92.6
Female	37	7.4
Ocular manifestations		
Yes	39	7.8
No	461	92.2
Comorbidities		
Yes	200	40
No	300	60
CT value		
≤30	471	94.2
≥30	29	5.8
WBC (white blood cell)		
≤11	477	95.4
≥11	23	4.6
CRP (C-reactive protein)		
≤15	263	52.6
16–50	133	26.6
51–100	56	11.2
≥100	48	9.6

Table 2 is showing a descriptive analysis of COVID-19 patients. The core body temperature was 37.3 ± 0.65 °C, CRP 32.9 ± 5.14, CT value 21 ± 5.14, age 44.7 ± 10.4 years, and hospital length of stay was 3.8 ± 5.27 days.

**Table 2.** Inflammatory and demographic variables.

Variable	Mean (SD)
Temperature (°C)	37.36 ± 0.65
WBC count	6.36 ± 2.4
Lymphocyte	1.6 ± 0.70
CT Value	21.89 ± 5.14
Age (years)	44 ± 10.4
Hospital stay	3.8 ± 5.2

Table 3 is showing the details of ocular manifestations in COVID-19 patients. The majority of our patients had hyperaemia (13 [33.3%]), followed by eye pain (9 [23.1%]), epiphora (8 [20.5%]), burning sensations (4 [10.3%]), and photophobia (2 [5.1%]) patients.

**Table 3.** Ocular manifestations.

Variables	No, number (percentage)	Yes, number (percentage)
Conjunctivitis	37 (94.9)	2 (5.1)
Hyperaemia	26 (66.7)	13 (33.3)
Epiphora	31 (79.5)	8 (20.5)
Photophobia	37 (94.9)	2 (5.1)
Eye pain	30 (76.9)	9 (23.1)
Burning sensation	35 (89.7)	4 (10.3)

Table 4 shows the comparison of ocular manifestations with other variables and their significance. There was no significant relationship between patients’ gender and occurrence of ocular manifestations (*p* = .43), comorbidities (*p* = .4), or CT value (*p* = .31).

**Table 4.** Correlations between variables and ocular manifestation.

Variable				*p*-Value
Ocular manifestation & gender	Male (number/percentage)		Female (number/percentage)	.43
	No	426/92.0	35/94.6	
	Yes	37/8.0	2/5.4	
Ocular manifestation & comorbidities					Comorbidities		
		No	Yes				
	No	276/92	185/92.5	.4			
	Yes	24/8	15/7.5				
Ocular manifestation & CT value	CT values						
	≤30		≥30	.31			
	No	433/91.9	28/96.6				
	Yes	38/8.1	1/3.4				

## Discussion

COVID-19 infection can involve all body organs and systems. Any part of the eye and ocular adnexa can get involved in COVID-19 patients. Ocular manifestations in these patients can either occur due to direct implantation of viral load in conjunctival mucosa [8] or through the haematogenous route to the posterior segment [9]. In our COVID-19 patients, 7.8% had symptoms related to the eyes. Literature mentions the incidence of ocular manifestations from 0.8% to 11.03% depending on the institution and area [1,2]. Sen et al. reported a higher incidence of up to 32% of COVID-19 patients [8].

Almazroa and colleagues, in their review, also reported 32% of the COVID-19 patients presented with ocular signs and symptoms [10]. Agrawal et al., in their review, reported that follicular conjunctivitis occurred in 7%, chemosis in 4.4%, conjunctival congestion in 10.89% of patients [11]. Wu et al. and Hang et al. reported equal percentages of ocular signs, mainly redness, dryness, ocular pain, foreign body sensation, discharge, itching, and follicular conjunctivitis [3,12].

In our patients, the most frequent ocular manifestation was conjunctival hyperaemia (33.3%), followed by eye pain (9 [23.1%]), epiphora (20.5%), burning sensation (10.3%), and less frequent were conjunctivitis and photophobia in 5.1% of patients.

Zhang et al. reported conjunctivitis to be the most frequent ocular manifestation in their population [13]. Tostman et al. reported ocular pain as the most frequent ocular symptom in COVID-19 patients [14]. Xu et al. reported itching as the prime ocular symptom, whereas Karimi et al. described foreign body sensation as the most frequent symptom in COVID-19 patients [9,15].

Significant changes occur in the immune and coagulation systems in COVID-19 patients. It is also possible that the virus can spread to the eye by haematogenous route, thus involving the eyes’ posterior segment (i.e. retina, choroid, and vitreous) though it is not known to be very common [16]. In a small cohort of asymptomatic COVID-19 positive patients, a group of authors has reported few retinal lesions in the form of cotton-wool-spot-like lesions and retinal microhemorrhages in 33% of patients [17]. There are also many case reports and publications on ophthalmic manifestations in COVID-19 patients and on the association of COVID-19 with different parts of the eye and its adnexa starting from the conjunctiva, episcleral tissue, sclera, extraocular muscles, orbit, posterior segment, optic nerve, and the visual pathways [18–20]. This study has a few limitations. Ophthalmologists were not involved in a comprehensive evaluation of ophthalmic symptoms of these patients, this study being retrospective in nature. The other important limitation of our study is the unavailability of data on the posterior segment and the retinal involvement.

To the best of the authors’ knowledge, this is the first study showing no correlation between patients’ gender, comorbidities, and occurrence of ocular manifestations in COVID-19 patients.

## Conclusion

The ocular manifestations were in the lower range in our patients compared to the literature. The patient’s gender and comorbidities did not correlate with the occurrence of ocular signs and symptoms. These patients did not necessarily have fever or leukocytosis upon presentation. Early detection and proper use of personal protective equipment could aid in the prevention of COVID-19 transmission.

## Data Availability

All generated data is included in this published article. All authors read and approved the final manuscript.
